# Mechanisms and Therapeutic Strategies to Overcome Enzalutamide Resistance in Advanced Prostate Cancer

**DOI:** 10.17161/sjm.v2i4.24058

**Published:** 2025-08

**Authors:** Xinyi Wang, Zhiguo Li, Xiaoqi Liu

**Affiliations:** 1 Department of Toxicology and Cancer Biology, University of Kentucky, Lexington, Kentucky 40536, USA; 2 Markey Cancer Center, University of Kentucky, Lexington, Kentucky 40536, USA

**Keywords:** prostate cancer, Castration resistance, Enzalutamide resistance

## Abstract

Enzalutamide, a second-generation androgen receptor (AR) inhibitor, has significantly improved survival outcomes in patients with metastatic castration-resistant prostate cancer (mCRPC). However, resistance to enzalutamide emerges in nearly all patients, posing a major clinical challenge. This review comprehensively examines the multifaceted mechanisms underlying enzalutamide resistance, including AR amplification, point mutations, splice variants such as AR-V7, and the activation of bypass signaling pathways, including glucocorticoid receptor signaling, PI3K/Akt/mTOR, and Wnt signaling. Additionally, the tumor microenvironment, lineage plasticity, and neuroendocrine differentiation contribute to therapy resistance through immune evasion and epigenetic reprogramming. We further discuss promising therapeutic strategies aimed at overcoming resistance, including combination treatments with PARP inhibitors, epigenetic modulators, and next-generation androgen receptor (AR) inhibitors. Biomarkers and personalized medicine approaches increasingly guide ongoing clinical trials to optimize treatment efficacy. This review also highlights drug repurposing as a practical avenue for targeting noncanonical vulnerabilities in resistant prostate cancer. Together, these insights provide a foundation for developing more durable and individualized treatment strategies for patients with enzalutamide-resistant metastatic castration-resistant prostate cancer (mCRPC).

## Introduction

Enzalutamide has emerged as a pivotal therapy in the management of metastatic castration-resistant prostate cancer (mCRPC), offering substantial improvements in patient survival and quality of life. Despite its clinical benefits, most patients ultimately experience disease progression due to the development of acquired resistance. Understanding the underlying mechanisms of resistance is critical to improving therapeutic outcomes. In this review, we examine the diverse molecular and cellular processes that contribute to enzalutamide resistance, including alterations in the androgen receptor signaling axis, activation of alternative oncogenic pathways, and modulation of the tumor microenvironment. Researchers also explore innovative therapeutic strategies designed to circumvent resistance, ranging from combination regimens with DNA repair inhibitors and epigenetic modifiers to emerging approaches targeting lineage plasticity and immune evasion. Finally, we discuss ongoing clinical trials and the growing emphasis on biomarker-driven, personalized treatment paradigms aimed at overcoming resistance and enhancing the durability of response.

## 1. Enzalutamide and Its Role in mCRPC

First, prostate cancer (PCa) was described in 1853 [1]. Men who were diagnosed with PCa and metastases would have a 1–2-year life span since then [2]. However, the first systemic approach to treat prostate cancer was invented by Huggins and Hodges in 1941, which demonstrated the effectiveness of androgen deprivation therapy (ADT) in treating metastatic prostate cancer [3]. This work builds up the foundation for investigating the fundamental role of androgens in PCa progression and the eventual development of resistance to ADT, now known as CRPC [4]. The average survival for patients with mCRPC is typically between 9 and 13 months, highlighting the need for effective treatment strategies. Despite significant advances in treatment options, mCRPC remained a major clinical challenge due to its unsatisfactory prognosis and limited survival rates until Enzalutamide was invented [5].

**Figure 1.**
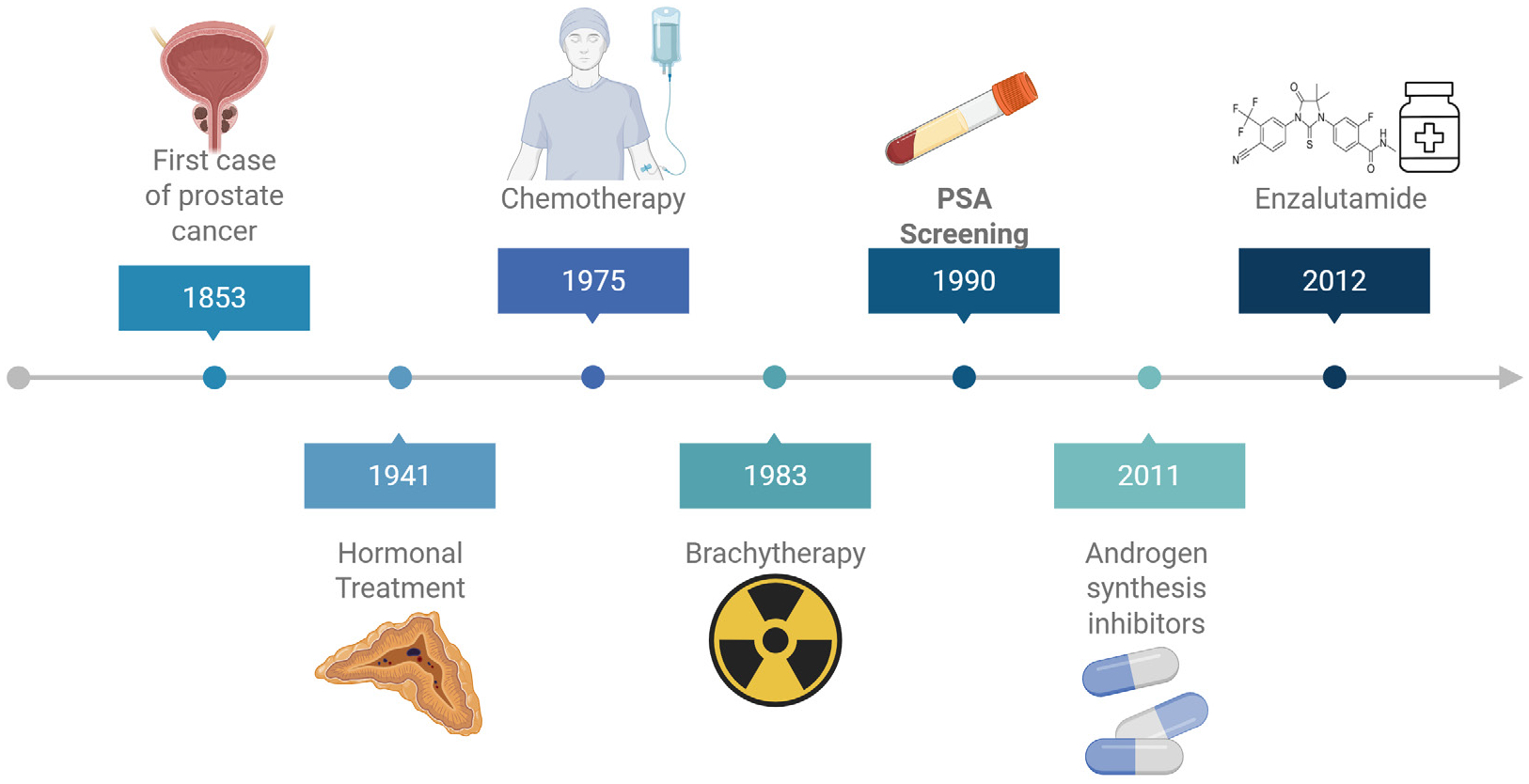
Milestones in the History of Prostate Cancer Diagnosis and Treatment This timeline outlines key developments in prostate cancer diagnosis and treatment from the 19th century to the present day. The first documented case of prostate cancer was recorded in 1853. Major therapeutic advances began with the introduction of hormonal treatment in 1941. Chemotherapy emerged in 1975, followed by brachytherapy in 1983. The 1990s marked a significant breakthrough in diagnosis with the adoption of PSA (prostate-specific antigen) screening. More recent advancements include the development of androgen synthesis inhibitors in 2011 and the approval of enzalutamide, a second-generation androgen receptor inhibitor, in 2012. These milestones reflect the evolving understanding and management of prostate cancer.

It has taken more than a century for researchers to develop a systematic therapeutic strategy for prostate cancer (PCa). (Figure 1) Enzalutamide was approved by the FDA in 2012 for patients with metastatic castration-resistant prostate cancer (mCRPC) who had previously received docetaxel [6, 7]. Upon Enzalutamide treatment, a final 5-year survival analysis has demonstrated significant clinical benefits in metastatic castration-resistant prostate cancer (mCRPC) [8]. In addition, Enzalutamide is well-tolerated and improves health-related quality of life (HRQoL) in patients with metastatic castration-resistant prostate cancer (mCRPC) [9]. Studies have shown that it delays the deterioration of HRQoL and reduces the progression of pain. Mechanically, Enzalutamide acts by inhibiting androgen receptor signaling, which is crucial for the growth and survival of prostate cancer cells [10]. It impairs the nuclear translocation of the androgen receptor and its binding to DNA, thereby reducing the expression of androgen-dependent genes.

However, resistance to enzalutamide leads to treatment failure in PCa, resulting in disease progression and reduced survival rates [11]. Studies have shown that resistance can significantly shorten the duration of the AR inhibition response. Furthermore, Enzalutamide resistance often results in cross-resistance to other androgen receptor-targeting therapies [12]. This cross-resistance limits the effectiveness of subsequent treatments, making it challenging to manage disease progression. The emergence of resistance not only affects survival but also impacts quality of life. As the disease progresses, patients may experience increased pain, fatigue, and other symptoms that reduce their overall well-being [13]. These investigations highlight the importance of studying Enzalutamide resistance in advanced prostate cancer to mitigate the impact on patients’ overall well-being.

## 2. Mechanisms of Enzalutamide Resistance

Enzalutamide resistance in prostate cancer (PCa) involves several complex mechanisms that enable cancer cells to evade the therapeutic effects of androgen receptor (AR) inhibition. Here, we summarize the key mechanisms.

### 2.1 AR overexpression, AR mutations, and AR splice variants

Hoefer *et al*. discovered that elevated AR expression exists in Enzalutamide-resistant prostate cancer cell lines [14]. In this paper, the authors found that the overexpression of AR diminishes the inhibitory effects of enzalutamide.

Specific AR mutations, such as H875Y, F877L, and T878A/S, were frequently identified after Enzalutamide resistance developed [15–17]. All these mutations were found in the AR ligand-binding domain (LBD), which potentially converts enzalutamide from an antagonist into an agonist, promoting tumor growth [18]. These mutations are observed in approximately 15% of patients with progressive metastatic castration-resistant prostate cancer (mCRPC) on enzalutamide therapy [19].

Besides AR mutations, AR splice variants, particularly AR-V7, also play a central role in Enzalutamide resistance [20]. AR-V7 expression in tumor cells is associated with shorter survival. These variants lack the ligand-binding domain, which can be targeted by enzalutamide. Because the variants retain AR constitutive functional activity, patients who harbor these variants are unlikely to respond to Enzalutamide.

### 2.2 Bypass Signaling Pathways in Enzalutamide Resistance

Signaling pathways that bypass androgen signaling are a significant mechanism contributing to enzalutamide resistance in prostate cancer. Here are some key pathways involved.

Glucocorticoid Receptor (GR) upregulation was discovered in enzalutamide-resistant prostate cancer cell lines and mouse xenografts [21]. Arora et al. found that elevated GR expression has been confirmed in both preclinical models and clinical samples of enzalutamide-resistant prostate cancer. In addition, GR has shared targets with AR, which enables the activation of AR target genes by an alternative pathway of GR, bypassing androgen signaling entirely. Besides the elevation of GR, sustained cortisol levels during enzalutamide therapy further amplify GR signaling, contributing to resistance [22].

Studies of the PI3K/Akt/mTOR pathway have been conducted extensively in human cancer [23]. One of the in *vivo* studies, using the PbCre; PtenloxP/loxP mouse model, showed a decreased AR level and its activity [24]. Inhibiting the PI3K pathway rescues AR protein levels. Inhibition of AR increases the active form of Akt, which can activate a potent oncogenic pathway [25]. Further research established that AR inhibition reduces FKBP5 and destabilizes PHLPP, an enzyme that inactivates Akt [26]. As a result, the accumulation of the active form of Akt results in tumor progression [24].

Wnt5A is a key ligand for noncanonical Wnt signaling, independent of the β-catenin pathway. Increased noncanonical Wnt signaling, mainly through Wnt5A, has been observed in enzalutamide-resistant cells [27]. A combination of ROCK inhibitor and Enzalutamide enhanced the efficacy of Enzalutamide.

Additional pathways, including autophagy and lineage plasticity, also contribute to enzalutamide resistance by allowing cells to adapt and survive under selective pressure [28, 29]. Nguyen et al. reported that AR blockage triggered an autophagic cascade. Depleting AMP-dependent kinase (AMPK), a cell stress sensor, inhibits autophagy and sensitizes PCa cells to enzalutamide-induced cell death [28]. Genomic aberrations in tumor suppressor genes, such as PTEN, RB1, TP53, and BRCA2, are frequently observed in biopsies from patients treated with enzalutamide. Loss of tumor suppressors TP53 and RB1 drives lineage plasticity, enabling prostate adenocarcinoma cells to switch to an AR-independent neuroendocrine phenotype [30]. This transformation is associated with increased expression of neuroendocrine markers, such as SOX2, and decreased expression of luminal cell markers. Neuroendocrine prostate cancer (NEPC) is highly aggressive and resistant to AR-targeted therapies, including enzalutamide [31, 32].

In summary, enzalutamide resistance in prostate cancer arises through a multifaceted network of mechanisms that enable tumor cells to evade AR-targeted therapy. These include alterations in the androgen receptor itself; such as overexpression, mutations in the ligand-binding domain, and the emergence of splice variants like AR-V7, which sustain AR signaling despite treatment [20]. In parallel, bypass signaling pathways, such as glucocorticoid receptor activation, PI3K/Akt/mTOR upregulation, and noncanonical Wnt signaling, provide alternative routes for tumor progression [24, 27]. Furthermore, cellular adaptations such as autophagy and lineage plasticity, often driven by the loss of tumor suppressors like TP53 and RB1, contribute to the development of therapy-resistant, aggressive phenotypes such as neuroendocrine prostate cancer [33]. As a result, understanding these mechanisms is essential for developing more effective combination therapies and advanced treatment strategies.

### 2.3 Tumor Microenvironment Modulation

Researchers have identified that enzalutamide-resistant prostate tumors frequently exhibit an immunosuppressive tumor microenvironment (TME) characterized by reduced infiltration of cytotoxic immune cells and altered cytokine expression profiles.[34] These changes impair anti-tumor immunity, allowing tumor cells to persist under androgen receptor (AR)-targeted therapy. In particular, the loss of immune surveillance has been linked to increased mesenchymal features and estrogen receptor (ER) expression, which together reduce the susceptibility of tumor cells to natural killer (NK) cell-mediated cytotoxicity [35, 36]. In enzalutamide-resistant models, fulvestrant, a selective estrogen receptor degrader, was shown to restore NK cell activity by enhancing tumor–NK cell conjugation [37]. This finding underscores the role of estrogen signaling in shaping an immune-suppressive microenvironment.

Additionally, the accumulation of immunosuppressive cell types, including regulatory T cells (Tregs) and myeloid-derived suppressor cells (MDSCs), further dampens immune responses in the resistant setting [38]. Checkpoint molecule expression is often elevated in resistant tumors, contributing to T-cell exhaustion and impaired anti-tumor activity [39]. These adaptations, driven by the TME, support enzalutamide resistance by enabling tumor cells to escape immune-mediated elimination.

Taken together, the immunosuppressive features of the tumor microenvironment actively promote resistance to enzalutamide by weakening immune pressure and facilitating the survival of tumor cells. Understanding these TME-driven resistance mechanisms offers a foundation for therapeutic strategies that combine AR-targeted therapy with immune-modulatory approaches.

### 2.4 Lineage Plasticity and Neuroendocrine Differentiation

Researchers have discovered that the loss of tumor suppressors TP53 and RB1 is a hallmark of lineage plasticity and neuroendocrine features [20, 40–42]. The loss of these two key tumor suppressor genes increases SOX2, which enhances the transition of prostate adenocarcinoma cells into stem-like or neuroendocrine cell states [43]. In addition, other stemness-associated transcription factors, such as ONECUT2 and E2F1, are involved [43, 44]. TCF4 overexpression in prostate cancer cells directly induces mRNA and protein levels of CHGA, neuron-specific enolase (NSE), and PTHrP, even in the absence of Wnt pathway activation [45]. These changes enable tumor cells to bypass AR dependency and adopt enzalutamide therapy-resistant phenotypes.

Moreover, alterations in the epigenetic landscape of prostate cancer have been identified as key drivers of lineage plasticity and neuroendocrine differentiation, contributing to the development of enzalutamide resistance [46]. The loss of methylation at SOX2, POU3F2, and ASCL1 promotes cancer cells’ neuroendocrine and stem-like transcriptional programs [47].

Collectively, lineage plasticity and neuroendocrine differentiation (NED) have also emerged as critical adaptive mechanisms driving resistance to enzalutamide. These alterations allow prostate tumor cells to escape dependence on AR signaling. AR-independent lineage, particularly NED phenotypes, exhibit aggressive and therapeutic refractoriness.

## 3. Ongoing Clinical Trials and Personalized Medicine Approaches

Overcoming resistance to enzalutamide is a critical challenge in the treatment of metastatic castration-resistant prostate cancer (mCRPC). In response, several ongoing and planned clinical trials are investigating innovative therapeutic strategies that combine enzalutamide with other agents to enhance efficacy and delay or prevent the onset of resistance (Table 1). These trials not only explore new drug combinations but also set the stage for personalized approaches based on the molecular profiles of individual tumors.

One of the most notable clinical efforts is the TALAPRO-2 trial, a Phase 3 study evaluating the combination of talazoparib, a PARP inhibitor, with enzalutamide in patients with Homologous Recombination Repair (HRR)-deficient metastatic castration-resistant prostate cancer (mCRPC). The results have been promising, with the combination showing significant improvements in both overall survival and radiographic progression-free survival compared to enzalutamide alone. These benefits are especially pronounced in patients with HRR gene alterations. Based on the final overall survival data presented at ASCO GU 2025, this combination is now being considered for broader use, including in patients without HRR deficiencies, highlighting its potential impact beyond genetically defined subgroups [48].

Complementing this approach is the MEVPRO-2 trial, a large-scale Phase 3 study assessing the efficacy of mevrometostat (PF-06821497), an epigenetic modifier targeting EZH2, in combination with enzalutamide. This trial enrolls AR pathway inhibitor (ARPI)-naive metastatic castration-resistant prostate cancer (mCRPC) patients and randomizes them to receive either combination therapy or enzalutamide with a placebo. The rationale is grounded in the hypothesis that EZH2 inhibition may prevent or delay epigenetic reprogramming events that contribute to resistance. With approximately 900 patients enrolled, this trial represents a significant effort to determine whether targeting chromatin regulators can enhance the durability of AR-targeted therapies [49].

Another promising trial is the combination of enzalutamide with masofaniten, a novel androgen receptor-targeting agent. Masofaniten is an androgen receptor (AR) N-terminal domain (NTD) inhibitor. Comparing Masofaniten with Enzalutamide, it disrupts AR transcriptional activity and can avoid AR ligand binding domain mutations as well as splice variants [50]. This approach is being evaluated in a Phase 1/2 trial involving mCRPC patients who have not previously received second-generation androgen receptor (AR) inhibitors [51]. Preliminary data from this trial suggest encouraging prostate-specific antigen (PSA) response rates and a manageable safety profile. The trial has now entered global Phase 2 expansion to assess further its efficacy and safety in a broader patient population, signaling strong momentum toward potential clinical adoption.

In addition to these trials, future directions in mCRPC treatment are increasingly oriented toward personalized medicine approaches. A key component of this strategy is the development of biomarkers [52]. Identifying reliable biomarkers, such as AR-V7 expression, can help predict which patients are likely to develop resistance to enzalutamide and which therapies they are most likely to benefit from [53]. Advances in genomic and proteomic profiling are enabling more refined patient stratification, thereby enhancing the precision of treatment selection [54, 55]. Further precision medicine trials are being designed with biomarker-driven treatment allocation to build on this. These trials aim to tailor therapeutic interventions to the molecular characteristics of individual tumors, improving clinical outcomes and reducing unnecessary exposure to ineffective treatments. This evolution in trial design reflects a growing recognition that “one-size-fits-all” strategies are insufficient in the context of complex resistance mechanisms.

Several next-generation combination therapies are also under development. For example, novel AR inhibitors, such as darolutamide, are being engineered to target AR mutants resistant to enzalutamide more effectively, offering a viable path forward for tumors harboring difficult-to-treat mutations [56]. Furthermore, integrating immunotherapies and targeted agents into treatment administration, for example, combining enzalutamide with immune checkpoint inhibitors or additional PARP inhibitors, may simultaneously enhance anti-tumor responses by attacking resistance through multiple biological pathways [34].

## 4. Discussion and Future Directions

**Figure 2.**
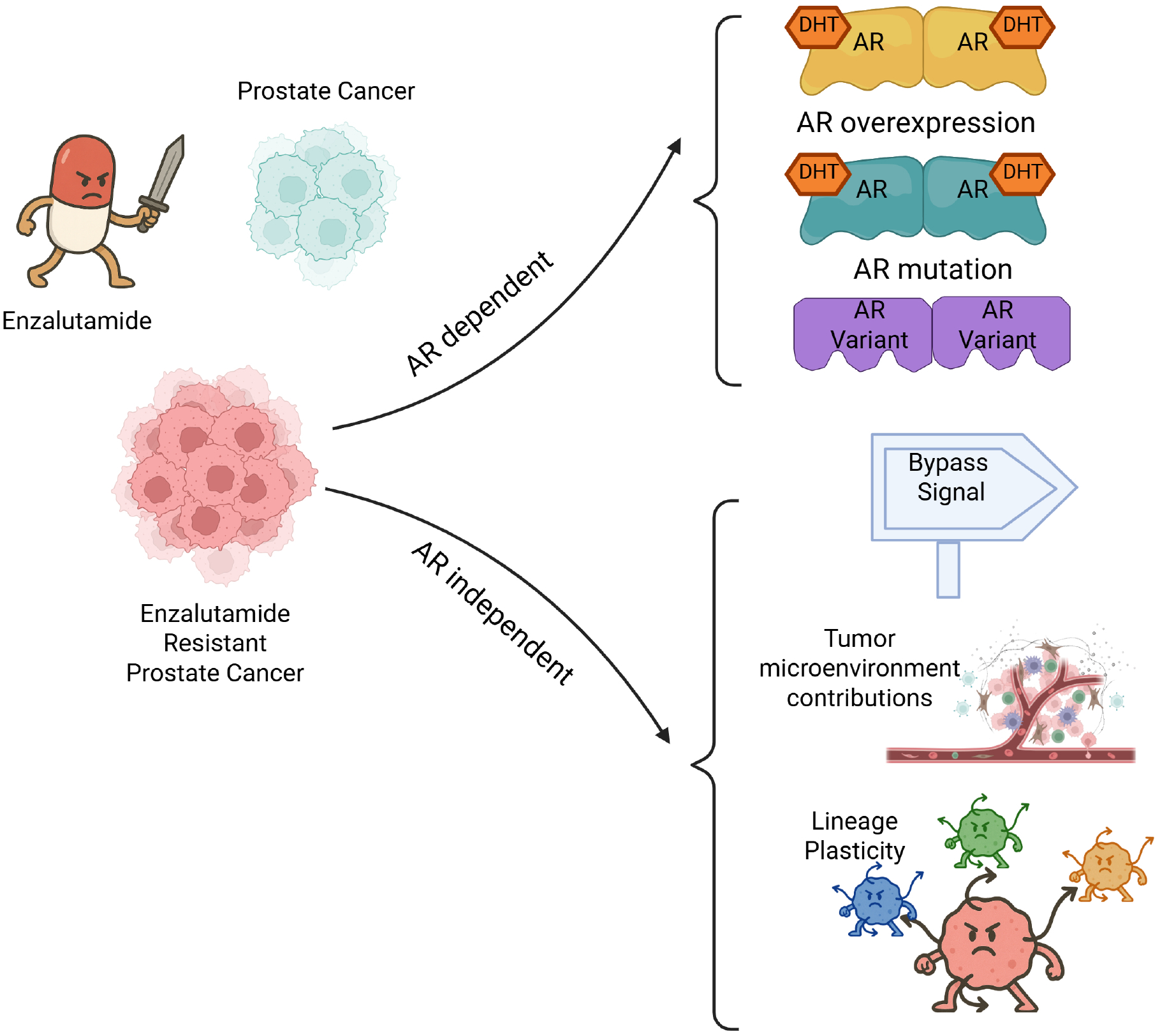
Mechanisms of Enzalutamide Resistance in Prostate Cancer This diagram illustrates the mechanisms underlying resistance to enzalutamide, a second-generation androgen receptor (AR) inhibitor used to treat prostate cancer. Resistance can emerge through AR-dependent or AR-independent pathways. In AR-dependent mechanisms, cancer cells continue to rely on AR signaling despite enzalutamide treatment, often due to AR amplification, AR mutations, or the emergence of constitutively active AR splice variants. In AR-independent mechanisms, cancer bypasses the AR axis through alternative signaling pathways, changes in the tumor microenvironment, or lineage plasticity, where prostate cancer cells adopt other cell identities to evade AR-targeted therapies. These pathways highlight the complexity of treatment resistance and the need for multifaceted therapeutic strategies.

The mechanism of Enzalutamide resistance in prostate cancer is multifaceted, involving both AR-dependent and AR-independent processes (Figure 2). These include AR amplification, point mutations, and the emergence of AR splice variants such as AR-V7, which allow sustained signaling despite AR inhibition [57]. Additionally, bypass signaling through glucocorticoid receptor activation, PI3K/Akt/mTOR pathway dysregulation, and noncanonical Wnt signaling enables tumor progression in an AR-independent manner [25]. Epigenetic reprogramming and lineage plasticity further enhance tumor cell survival under therapeutic pressure, while the immunosuppressive tumor microenvironment promotes immune evasion and therapeutic resistance [58–60].

The interplay among these mechanisms underscores the importance of developing multifaceted therapeutic strategies. Combination therapies targeting DNA repair deficiencies, chromatin modifiers, or immune checkpoint pathways have shown promise in preclinical and clinical studies [61–63]. Biomarkers such as AR-V7, SOX2, and TCF4 help identify patients at higher risk of resistance and guide personalized treatment selection [45, 64]. As clinical trials progress, the integration of genomic and proteomic profiling into therapeutic decision-making will become essential for tailoring treatments to tumor-specific vulnerabilities.

In this context, drug repurposing has emerged as a practical and cost-effective strategy to accelerate the development of new therapies for enzalutamide-resistant prostate cancer. Repurposed drugs, already approved for other indications, offer the advantage of well-characterized safety profiles and established pharmacokinetics, significantly reducing the time and cost required for clinical deployment. Recent examples include the use of Artesunate, an antimalarial agent, to degrade c-Myc and restore enzalutamide sensitivity in resistant prostate cancer cells [57]. Similarly, fulvestrant, a selective estrogen receptor degrader initially developed for breast cancer, has shown the potential to reverse immune escape in enzalutamide-resistant tumors by restoring NK cell-mediated cytotoxicity [37]. The rationale for drug repurposing lies in targeting noncanonical vulnerabilities, such as metabolic reprogramming, lineage plasticity, and immune evasion, that are not directly addressed by conventional AR-targeted therapies. Additionally, mining large-scale pharmacogenomic databases and integrating patient-derived organoid models with high-throughput drug screening may further identify compounds with efficacy against resistant phenotypes.

Overall, overcoming enzalutamide resistance will require a systems-level approach that integrates molecular diagnostics, biomarker-guided therapies, rational drug combinations, and strategic repurposing of existing agents. As research advances, these integrated strategies have the potential to significantly improve outcomes for patients with advanced prostate cancer and redefine treatment paradigms for enzalutamide-resistant prostate cancer.

## Figures and Tables

**Table 1.** Ongoing Clinical Trials Targeting Enzalutamide Resistance in mCRPC Several clinical trials are actively exploring combination strategies to overcome enzalutamide resistance in metastatic castration-resistant prostate cancer (mCRPC). These studies target diverse mechanisms, including DNA repair defects, epigenetic reprogramming, and non-ligand-binding domains of the androgen receptor. Early results show promise in extending survival, enhancing response rates, and addressing resistance across genetically diverse patient populations. Biomarker-driven stratification is increasingly used to guide therapy selection and optimize treatment efficacy.

Trial	Target	Combination	Patients Population	Key Outcome
TALAPRO-2	HRR pathway	Enzalutamide + Talazoparib	HRR-deficient mCRPC	↑ PFS & OS
MEVPRO-2	Epigenetic (EZH2)	Enzalutamide + Mevrometostat	ARPI-naive mCRPC	Ongoing
Masofaniten	AR-NTD	Masofaniten ± Enzalutamide	mCRPC, ARSI-naive	PSA ↓, tolerable
